# Recently Appointed Consultants

**Published:** 1969-07

**Authors:** 


### RECENTLY APPOINTED CONSULTANTS

**DR. DONALD SHORT,** a newly appointed Consultant Anaesthetist to the United Bristol Hospitals and Winford Orthopaedic Hospital, comes to us after a career at The London and The Middlesex Hospitals. His main interests are cardiac anaesthesia and intensive care, and he is to be full time at present.

He is married, has two children, and is looking for a house just outside Bristol on the south-western side.

He has recently joined the British division of The Royal Navy Reserve.

**DR. PETER DUNN** has recently been appointed Lecturer in Child Health and Consultant in Neonatal Paediatrics at Southmead Hospital and the Royal Hospital for Sick Children. He received his training in Cambridge, Birmingham, Warwick, Bristol and San Francisco. His interests include the study of foetal growth and adaptation at birth, congenital deformities and haemolytic disease of the newborn. He is married, has three children and lives in Westbury-on-Trym. His hobbies include tennis, golf and sailing.

**MR. ROGER FENELEY** has recently been appointed Consultant Urologist to the United Bristol Hospitals and South West Regional Hospital Board. He is a Bristolian by birth and was educated at Clifton College. At Cambridge he gained a Boyd Scholarship at Corpus Christi College, and First Class Honours in the Natural Science Tripos. He completed his clinical studies at Guy's Hospital and had a broad education in General Surgery with posts in London, Bristol, and Guildford, before returning to Guy's as Surgical Registrar. He came to Bristol as Senior Registrar in 1966 and has been working in the Department of Urology for the past two years. He gained the F.R.C.S. in 1962 and M.Chir. in 1967. His main interests are concerned with studies of renal function and bladder function in obstructive uropathy.

He is married and has three children who have temporarily curtailed his aspirations on the golf course.

**DR. BETTY BROWNELL,** a graduate of Trinity College, Dublin, has recently been appointed Consultant Neuropathologist, in the Bristol Clinical Area. She will be full-time, based at Frenchay Hospital, with one session at the Bristol Royal Infirmary, and hopes in time to build up a reference service in neuropathology for the South Western Region.

Dr. Brownell has worked for the past 12 years in Oxford, where she was a Research Fellow and lecturer at the Radcliffe Infirmary. Within the sphere of neuropathology, her particular interests are neuromuscular disease, and disorders of the peripheral nervous system, and she hopes to carry out research on muscle enzyme histochemistry in neurogenic muscle atrophies.

Dr. Brownell will be living in Westbury-on-Trym. For recreation, she enjoys outdoor pursuits, including birdwatching.

